# Gleanings from Our Exchanges

**Published:** 1873-11-01

**Authors:** 


### Gleanings from Our Exchanges.

M. Peter's Clinique. Stricture of the Lower part of the Intestines.—In a lecture dilivered in the Hopital Saint Antoine on the 25th July last (*Le Mouvement Medical*, 9th August), Dr. Peter mentions the case of a man who asked to be taken in on Saturday, on account of a large abdomen, which much incommoded him. Distension of the abdomen was noticed, with meteorism; and Dr. Peter first of all diagnosed internal strangulation.

On the morrow he ascertained that the patient had been subject to constipation for some three days; and he had no evacuation, although certain purges had been given. Upon this, two drops of croton oil were given in the form of a pill; but the patient vomited these, and had no stool as result. On Monday there was vomiting of yellowish matter, stercoraceous, so that the diagnosis was confirmed. But what was the material lesion, was the question.

When the belly was attentively explored, it was found to be uniformly distented, and the percussion note everywhere tympanitic. There was serous effusion in the pelvic cavity, dullness and pasty thickening in the sides, and this pointed to retention of fæcal matters in the large intestine. After this examination the seat of the obstacle was easily determined. When it exists at the upper part, the tympanitic condition of the belly is general, but the flancs are not dull; that is to say that the ascending and descending colons, when empty, are covered by loops of the small intestine distended by gas, which give sonorous character to the sides and iliac fossæ. "I thought, then, that the obliteration had its seat at the lower part of the large intestine; but exploration by the rectum did not permit me to get as far as the obstacle."

"What, then, was the diagnosis of the occlusion? According to information given, I learn that two years ago this man had a slight dysentery, lasting from September 10 to October 3, which is called Parisian dysentery, that is to say, a benign disease. For do not forget, gentlemen, that there is a very notable difference between the dysentery of our country and that of warm climates. The latter offers the gravest lesions, characterized by ulcers of the intestinal mucous membrane, which are destroyed and expelled, perhaps latterly turned into scars, whose retraction is capable of causing strictures."

"On the other hand, the general condition of the patient, the good health he has preserved until quite lately, would appear to exclude the idea of a cancer of the large intestine; so that the diagnosis of the lesion which caused the suffering remained undecided between an intestinal occlusion from stricture or from cancer.

"However, at the autopsy, we found an epithelial tumor seated at the juncture of the iliac curvature with the rectum.

"Now, gentlemen, it is not indifferent to make the diagnosis, however uncertain this may be, since the nature of any surgical intervention may be influenced by it, and gastrotomy or enterotomy may be performed. On the other hand, if the lesion exist in a portion of the small intestine, and be due, for instance, to a false membrane which strangles the intestine externally, in this case gastrotomy may be performed, in order to seek out and get rid of the strangulation. I may here allude to a patient in my wards on whom M. Duplay practiced gastrotomy for the purpose of getting rid of an obstacle on which he did not chance to fall, perhaps because his incision was not large enough.

"On the other hand, if the obstacle be seated very far down, enterotomy is absolutely necessary; and this is what was practiced by Dr. Ledentu on our patient, in whom an opening was made in right iliac region. In such a case ought we to make a large or a small incision?

"I have seen this operation twice practiced by MM. Nelaton and Dolbeau. M. Nelaton had not, after the operation, any subacute peritonitis, the absence of which was certainly due to the very intimate apposition between the abdominal wound and the incision in the side. That surgeon made an incision of two centimetres in length, which he found even too great; and then punctured with the bistoury. And Dr. Dolbeau, like Nelaton, followed the same plan, and had no peritonitis, and the patient got well.

"In our patient, the peritonitis which carried him off in twenty-four hours, appears to me to have been due to infiltration of fæcal matters between the wall of the intestine and the abdominal wall."—*The Doctor.*

The Tragic Death of Obermeier.—Prof. Virchow devotes the following lines to the memory of his late assistant, Dr. Obermeier, who fell a victim, as is well known, to experimentation upon himself in seven hours after injecting the blood of a cholera patient into his own body. We extract literally from the *Berliner National Zeitung:*

"*Berlin, Aug.* 24.—Your paper this morning, in an article upon the deceased Dr. Obermeier, denies the statement that he made an injection of cholera blood into his own body. I am unable to discover the grounds which prompted your correspondent to make this negation. So far as I know, Dr. Obermeier communicated with none of his fellow-physicians upon this subject, but he did let fall some observations to his female nurse, while in full consciousness, that seem to leave no room for doubt that he did make this experiment. His peculiar disposition also lends it additional credibility. He was my assistant during a severe epidemic of typhus fever, and in all that period never expressed the least personal fear. He passed through the worst epidemics of small-pox without being moved in the least. And only eight days ago he visited me and remarked, with a cheerful face, that he had the cholera now, but would soon be over with it. Unfortunately, it is quite impossible to ascertain positively whether Dr. Obermeier died after an injection of cholera blood or not. Yet he had opportunity enough, without injection, to become infected with cholera 'germs.' May we not lack, in the future, individuals so free from all personal consideration, so devoted to scientific investigations, and so independent in their views and observations as Obermeier. R. Virchow."
—*The Clinic.*

Harvard Medical School.—C. Ellis, M. Dean of the Medical Faculty of Harvard University, takes, in a letter to the Philadelphia *Medical Times*, a very hopeful view of the success of the Harvard Medical School, and states that the receipts from students were only $3,600 less in 1871–72 than in 1870-71. One hundred and fifty students, under the new plan, gives a considerably larger revenue to the school than three hundred did under the old. He thus concludes: "If five or six of the other leading medical schools in the country would join the Harvard school in making the changes in the system of medical instruction, which Dr. Stille and all other intelligent physicians who honor their profession admit to be desirable, there would be no question in any one's mind about the success of the reform."

Dr. Gubler on Chloral.—Chloral, says Gubler (*J. de Chemie et de Phar.*, Sept.), renders every day important services in neuralgia, hepatic colic, cancer, gout, and pleurodynia. He finds chloral useful in whooping-cough. Chloral is but of slight use in chorea, and is of no use in hydrophobia. It is of no service in epilepsy or puerperal eclampsia. In tetanus it has been proved useful, first of all by Dr. Verneuil; but Gubler had two unsuccessful cases in which it was tried. According to Beck's statistics, out of 36 cases of tetanus treated by chloral there were 21 cures and 15 deaths. Chloral is evidently an hypnotic in a great number of cases of mental alienation, characterized by exaltation, talkative or furious delirium, suicidal monomania, or homicidal mania. The calm which it gives to the agitated patients renders them able to do without constraint. The agent, however, has sometimes been abused. The chloral, however, is contra-indicated in those mental affections which are accompanied with cerebral depression, debility, and especially where the circulation languishes and the nutrition is defective. It is, on the contrary, especially to be recommended in the acute, agitated, and violent forms, and notably in acute delirium, in the nervous delirium of fever, and of feverish inflammations, in hysterical insanity, and in delirium tremens. Dr. Gubler has found it very useful in the last-named disease. In this disease chloral equals in power the opium so justly praised, and is more rapid in its action. Gubler often combines the two remedies. Chloral is often useful as a hypnotic in fever; and this is, indeed, its powerful proportion in the course of bronchitis or pulmonary consumption.—*The Doctor.*

Strangulated Hernia and the Aspirator.—Another of these hernia cases was discovered about three hours after the patient had been admitted to the hospital for some other difficulty. In her journey to the hospital the hernia had come down enormously and become strangulated. The hernial tumor was as large as a child's head. It was hardly possible to determine what kind of hernia it was, the tumor, when freed, was of such size and tension. Taxis was unavailing, and probably would have been equally unavailing had a correct diagnosis been made, and taxis been made in the proper direction. Taxis was made in the proper direction for the reduction of an *indirect* inguinal hernia, but the sequel proved it to be a *direct* inguinal hernia. This is a mistake not so infrequently made as may be supposed. Taxis in this case being unsuccessful, the tumor was aspirated, and fully ℥ viij. of serum drawn off. The gut was not aspirated. The removal of this quantity of fluid rendered the tumor sufficiently flaccid to permit a much more easy and extended manipulation, assisted in arriving at a correct diagnosis, and by a little careful and well-directed handling the hernia was reduced. An operation was probably avoided by means of the aspirator.—*N. Y. Med. Record.*

College Students in the Middle Ages.—An attendance of 3,000 students at any university, at the present time, would be thought exceedingly large. In the times of the Reformation, and earlier, however, an attendance many times as large was nothing unusual. For example, at the University of Bologna, in 1350, there were 13,000 students. A little over a century later, at the death of Charles VII., 25,000 students were in attendance at the University of Paris; and, still another century later, when Joseph Scaliger was a student, the number at this famous institution had increased to 30,000.

Even in England there was a similar flocking of students to the great University of Oxford. It is stated that in the time of Henry III., about the middle of the thirteenth century, the number of students at Oxford amounted to 30,000. Admitting that there is exaggeration in this statement, the actual number is certainly known to have been very great.

Perhaps at no university was there a larger, or so large, an attendance as at that of Prague, in the days of Huss. The fame of this University, and the privileges granted to all its members, drew students from all parts of Germany. When the privileges, granted to the Germans equally with the Bohemians, were withdrawn, many thousands of German students left the University. It has been estimated by some authorities that as many as 44,000 students forsook their accustomed places of learning. This estimate is doubtless too high. But, from all reliable accounts, it would appear that in 1409 there were from 30,000 to 40,000 students in the University of Prague.—*The Repository*, Nov., '73.

Dr. T. Bates proposes (*The Clinic*) to substitute meal for bran in fracture-dressings as an absorbent. The meal, he claims, is equal to the other dressing, and is much superior in that it packs better and holds the limb immovable in the apparatus.

Late dispatches announced that the illness of M. Nelaton had taken a favorable turn, and he was supposed to be convalescent; but on Saturday last he suffered a relapse, sank rapidly, and died the same night, in the seventy-sixth year of his age.—*Phil. Med. Times.*

Marini's Petrifactions.—In the Italian section of the Vienna Exhibition, Dr. Marini exhibits, among an assortment of human feet, hands, legs, arms, and busts, of shrivelled proportions and deep-brown color, a large, round plateau, evidently of hard and polished material, which has been likened to stale gelatine or potted boar's head. It is a conglomerate of specimens illustrative of an art invented by him—the petrifaction and mumification of human corpses. It was this very Dr. Marini who petrified Mazzini, and executed his work so well that the admirers of the arch-conspirator proposed to set up the corpse on the capitol, and save Italy the expense of a statue. The preparations are waterproof, and will take on high degrees of polish. His mumified specimens, by a process known to him alone, can be restored to their original size and elasticity; while the petrified ones are as hard, and possibly as durable, as granite. The top slab of the table is composed of muscles, fat, sinews, and glandular substance, all petrified together in a block, the surface of which has been planed and polished until it resembles marble. Certificates from Nelaton and other distinguished surgeons are attached to the specimen limbs, setting forth that the limbs in question had, for the satisfaction of the certifiers, been restored to their pristine softness and pliancy by Dr. Marini.—*Lancet.*—*Boston Med. Journal*, Sept. 18, '73.

